# Systemic Diseases and Biological Dental Implant Complications: A Narrative Review

**DOI:** 10.3390/dj11010010

**Published:** 2022-12-29

**Authors:** Luca Sbricoli, Elissar Bazzi, Edoardo Stellini, Christian Bacci

**Affiliations:** Unit of Dentistry, Department of Neurosciences, University of Padova, 35128 Padova, Italy

**Keywords:** dental implants, mucositis, peri-implantitis, systemic diseases

## Abstract

The relationship between periodontitis and such systemic disorders as diabetes, cardiovascular disease and obesity has been extensively investigated. There is less scientific evidence available, however, regarding the influence of systemic diseases on the risk of late failure of dental implant rehabilitation due to peri-implantitis. The aim of the present study was to review the literature on the role of several common systemic disorders (diabetes, obesity, cardiovascular disease, hypertension and osteoporosis) in the onset of peri-implantitis. A database search initially yielded 2787 studies of potential interest published up to 1 March 2022 (993 in PubMed; 908 in Web of Science; and 886 in Scopus). After removing 1190 duplicate articles and checking the titles, abstracts and full texts for relevance, 70 articles were selected for the present analysis. Only cohort, case-control studies and clinical case series were considered. Most of the literature concludes for no association between diabetes, cardiovascular disease, hypertension or osteoporosis and the risk of peri-implantitis. On the other hand, almost all the studies that investigated obesity as a risk factor for implant rehabilitation found a positive association between the two. Further longitudinal studies are needed to better understand the effects of systemic diseases on rehabilitation with dental implants.

## 1. Introduction

According to the report from the World Workshop on the Classification of Periodontal and Peri-Implant Diseases and Conditions in 2017, peri-implantitis is a pathological condition due to plaque forming in the tissues around dental implants. It is characterized by inflammation of the mucosa and a subsequent gradual loss of the underlying bone [1]. Judging from a meta-analysis conducted by Lee et al., weighted mean implant-based and subject-based peri-implantitis prevalences were 9.25% and 19.83%, respectively. Weighted mean implant-based and subject-based peri-implant mucositis prevalences were 29.48% and 46.83%, respectively [2].

Taken together, cardiovascular and chronic respiratory diseases, cancer and diabetes are responsible for almost 75% of deaths in Europe as a whole, and the principal cause of death in the 53 member states of the WHO European Region. Nowadays, it is well known that preventing and controlling such systemic diseases is also fundamentally important to the health of the oral cavity. The link between systemic diseases and periodontitis has been amply discussed in the literature. It has been established, for instance, that diabetic patients can experience twice as much tooth loss as healthy individuals [3]. Such patients are therefore more likely to need prosthetic rehabilitation measures, which increasingly involve dental implants [4].

In terms of the inflammatory and lipid profile of healthy recipients of dental implants with and without a diagnosis of peri-implantitis, there is evidence to suggest that those suffering from peri-implantitis have a low-grade systemic inflammatory state (higher circulating levels of white blood cells) accompanied by dyslipidemia (increased blood levels of total cholesterol and LDL cholesterol) [5].

Long-term follow-up studies point to peri-implantitis and cardiovascular disease sharing the same risk factors, both being associated with high lipid levels in the blood. To date, the literature remains inconsistent and controversial concerning the association between cardiovascular disease and biological complications affecting implants [6]. Since inflammation is common to both these conditions, it is important to further investigate this potential association in better-controlled studies on more numerous, and more homogeneous sample populations.

There are still very few publications on the influence of obesity on peri-implant tissue health, but an association between obesity and a higher risk of developing periodontitis had already emerged from previous systematic literature reviews [7,8,9,10,11]. One cross-sectional study identified a statistically significant correlation between obese patients’ waist circumference and the levels of proinflammatory cytokines in their peri-implant crevicular fluid, even when the peri-implant tissues were well preserved [12]. Chronic inflammation also induces oxidative stress, which could contribute to the onset of insulin resistance. This is an interesting finding because some studies suggest that insulin resistance can lead to the onset of diabetes type II, and to chronic periodontitis as well [13].

Published findings concerning any link between glycemic control and peri-implant disease are very mixed, but diabetes has been widely recognized as a factor capable of interfering with the postoperative healing process after the insertion of a dental implant. It emerged from a systematic literature review that rehabilitation with dental implants is a safe option if patients’ diabetes mellitus is well controlled [14]. There is still no accepted range of glycated hemoglobin (HbA1c) levels to indicate when diabetes is not controlled, however. More homogeneous reference parameters would be useful in order to clarify the impact of diabetes and glycemic control on peri-implantitis and dental implant failures.

Finally, as concerns osteoporosis, the World Workshop held in 2017 judged this disease to be significantly associated with a higher prevalence and greater severity of radiographically-evident alveolar bone loss. The mechanism behind this association is still not fully understood, however, and no clear links with any other clinical parameters of periodontitis have come to light. 

A drawback of previous research on the relationship between systemic diseases and peri-implantitis could lie in the scarcely consistent definition of peri-implantitis and its clinical indicators. 

The aim of the present narrative literature review was to investigate the role of several systemic disorders—diabetes, cardiovascular disease, hypertension, obesity and osteoporosis—in the late onset of complications and the failure of osseo-integrated dental implants.

## 2. Materials and Methods

A literature search was run using the following string in the following three search engines: PubMed, Web of Science and Scopus:

“(peri-implantitis OR peri-implant disease OR dental implant OR osseointegrated implant) AND (systemic disease OR dyslipidemia OR obesity OR metabolic syndrome OR diabetes mellitus OR hyperglycemia OR cardiovascular disease OR hypertension OR hyperlipidemia OR osteoporosis OR osteoporotic OR bisphosphonates OR antiresorptive agents)”.

Filters were used to select only studies in the English language, published until 1 March 2022. Duplicates were removed. Articles not conducted on human beings, those without an abstract and those with titles or abstracts judged inconsistent with the aims of the present review were also omitted; the final step was to remove the items with full text unavailable, single case reports, narrative reviews, systematic reviews, and meta-analyses. After reading the full texts, clinical studies diverging from the purposes of the present review were also rejected. The observational studies selected for review thus include cross-sectional, retrospective and prospective studies on series of clinical cases, case-control studies and cohort studies. The Covidence software was used as support.

## 3. Results

Applying the search string to the three search engines generated 2787 articles in all, including duplicates (993 in PubMed, 908 in Web of Science and 886 in Scopus). After removing 1190 duplicates, animal or in vitro studies and reading the remaining articles’ titles and abstracts, 242 studies were considered potentially eligible. After removing the case reports and systematic reviews and reading the full texts available, there were ultimately 70 studies judged to be consistent with the aims of our review.

**Figure 1:**
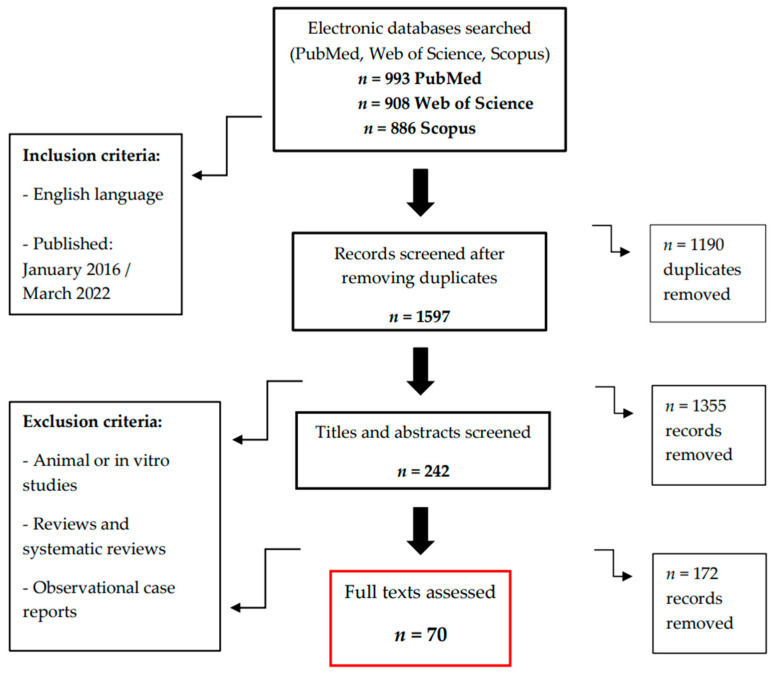
Flowchart of the decision-making process for the selection of the articles eligible for review.

The flowchart illustrates the decision-making process for the selection of the articles eligible for review (Figure 1). 

## 4. Types of Study Examined

Considering the articles by study design, 20 were prospective, 32 were retrospective, 13 were cross-sectional and for 5, the study design was not specified. All the articles retrieved from the electronic database search are listed in the Supplementary Materials (Tables S1–S4). 

Among the 20 prospective studies, 14 examined prediabetes and/or diabetes mellitus as a risk factor for the survival and/or success of dental implants, 6 considered osteoporosis/osteopenia and/or the use of anti-resorptive drugs, 3 investigated cardiovascular disease, and 1 focused on obesity. The 32 retrospective studies concerned prediabetes and/or diabetes mellitus in 23 cases, concerning osteoporosis and/or the use of antiresorptive drugs in 14, cardiovascular disease in 8, hypertension in 4, and obesity in 2. Among the 13 cross-sectional studies, 1 examined metabolic syndrome as a risk factor, 10 considered prediabetes and diabetes mellitus, 2 focused on cardiovascular disease, 3 on hypertension, and 3 on obesity. Finally, as concerns the five studies of unspecified design, three analyzed diabetes as a risk factor, one considered only prediabetes and one investigated both diabetes and prediabetes.

The main findings are outlined below, by type of systemic disease.

### 4.1. Diabetes and Prediabetes

In all, 51 studies were identified concerning the influence of prediabetes and diabetes mellitus type I or II on the onset of late complications in dental implants: 14 were prospective [15,16,17,18,19,20,21,22,23,24,25,26,27,28]; 23 retrospective [29,30,31,32,33,34,35,36,37,38,39,40,41,42,43,44,45,46,47,48,49,50,51]; 10 cross-sectional [52,53,54,55,56,57,58,59,60,61] and 5 of unspecified design [62,63,64,65,66]. Thirty-three of these studies found no association between the success or failure of dental implants and diabetes, while seventeen reported a positive correlation, and in one (cross-sectional) study the peri-implant inflammation parameters were better in patients with diabetes and hypertension than in healthy individuals [53] Articles were grouped by findings concerning diabetes as a risk factor for biological complications of dental implants (Table 1).

Four of the fifty-one studies [31,52,58,59] identified prediabetes as a risk factor for peri-implant inflammation. One of them [58] found statistically significant positive correlations both for advanced glycation end-product (AGE) levels with probing depth and marginal bone loss, and for marginal bone loss and diabetes. 

### 4.2. Obesity

Five [61,67,68,69,70] out of six studies identified a link between obesity and worse clinical and/or biomolecular parameters of peri-implant infection. Only one study found BMI associated with postoperative complications in dental implants, but not with implant failure; this association was not seen in patients with both diabetes and obesity, however [71]. Articles were grouped by findings concerning obesity as a risk factor for biological complications of dental implants (Table 2).

### 4.3. Cardiovascular Disease

As concerns a relationship between cardiovascular disease and dental implant complications or failure, this emerged in 13 studies in all (3 prospective, 8 retrospective, and 2 cross-sectional), with 11 of them [18,23,38,39,48,49,50,51,54,57,72] reporting no association with a greater risk of peri-implant disease. Only two studies [21,37] found worse outcomes for dental implants in patients with cardiovascular disease than in healthy individuals. Articles were grouped by findings concerning cardiovascular diseases as a risk factor for biological complications of dental implants (Table 3).

### 4.4. Hypertension

The seven studies identified (four retrospective and three cross-sectional) were almost unanimous in finding no evidence of hypertension being a risk factor for dental implant rehabilitation [44,46,47,53,54,57]. The only exception was one study reporting a higher rate of implant failure in hypertensive patients [48]. Articles were grouped by findings concerning hypertension as a risk factor for biological complications of dental implants (Table 4).

### 4.5. Osteoporosis and the Use of Antiresorptive Drugs

As for the influence of osteoporosis and/or the use of antiresorptive on the success of dental implant restoration, and the survival of the implants, most studies (n = 16) did not identify them as a risk factor [18,32,36,41,49,50,73,74,75,76,77,78,79,80,81,82]. On the other hand, four studies found that osteoporosis [38,46,51] and/or the use of antiresorptive drugs such as bisphosphonates [33] posed a risk to said success and survival, and also negatively affected implant marginal bone loss. One of these studies [46] reported only osteoporosis as a risk factor, not the use of bisphosphonates.

Articles were grouped by findings concerning osteoporosis (Table 5) and use of antiresorptive drugs (Table 6) as risk factors for biological complications of dental implants.

## 5. Discussion

This review pools recent evidence on possible associations between peri-implant health issues in the oral cavity and systemic diseases. Articles on this topic revealed a marked heterogeneity as concerns the type of study conducted and the results obtained, but the evidence to support such an association was limited or inconsistent for most of the systemic conditions considered.

### 5.1. Diabetes

The role of diabetes in the onset of complications affecting osseo-integrated dental implants has been investigated much more than that of the other systemic diseases considered here, but contrasting findings have emerged. Judging from our analysis of the literature of the last six years, only one in three studies identified an association between diabetes and peri-implant disease. As for prediabetes, there was one report [58] of AGEs possibly having an important influence on peri-implant inflammation in prediabetic and diabetic patients.

No statistically significant differences came to light in most of the studies focusing exclusively on implant failure or survival [17,18,32,39,44,45,48]; and two of these studies [18,44] boasted a remarkably long follow-up (31 years in one, 9 in the other). On the other hand, three studies [33,35,40] did identify diabetes as a risk factor for implant failure. One was a retrospective study by French et al. [40], who examined 10,871 implants with a long-term follow-up (22 years). It is worth emphasizing that some studies assessed ‘implant failure’ without specifying the criteria used to define it, or whether failures occurred early or late. One of the main limitations of these studies probably lies in that they failed to consider issues such as bleeding and probing depth, which are essential to a diagnosis of peri-implant disease [83].

Of the studies that only examined peri-implant marginal bone loss in relation to diabetes, four [36,38,43,64] reported finding no statistically significant differences vis-à-vis non-diabetic patients, whereas the other four studies that considered this parameter [24,47,49,55] did find an association. When comparing diabetics with nondiabetics, it would be important to assess patients’ oral hygiene levels as well. For example, one limitation of the study by Al-Zahrani et al. [24] could lie in their having failed to conduct a logistic regression analysis on how many times a day patients brushed their teeth (nondiabetics: 29% once a day and 71% twice a day; diabetics 58% once a day and 42% twice a day). Abduljabbar et al. [55] also mentioned the importance of patients with chronic hyperglycemia monitoring their HbA1c levels in order to remain within a controlled range to prevent peri-implant damage. The primary outcome of a recent systematic review and meta-analysis [84] pointed to a statistically significant association between peri-implant marginal bone loss and diabetes mellitus.

Out of six studies [34,54,57] examining the risk of peri-implantitis developing in diabetic patients, three [23,35,37] established a diagnosis of peri-implantitis based on different criteria from those adopted by the International Workshop of 2017. This is because they had been conducted earlier, but it is nonetheless a limitation. Another limitation of several studies [35,54] may concern the authors’ failure to measure patients’ blood sugar levels during the follow-up. Only one [54] of the above-mentioned six studies identified an association between diabetes and peri-implantitis. These results contrast with the findings of another recent review [85], which found that people suffering from diabetes mellitus had a twofold risk of developing peri-implant disease.

An interesting finding emerged from a study by Al-Askar et al. [30], as follows: diabetic patients’ inflammatory cytokine levels were influenced by their glycemic status rather than by any peri-implantitis. Alshahrani et al. [31] and Al Zahrani et al. [26] also found osseointegration impaired, more severe peri-implantitis and more frequent implant failures in diabetic patients with poor glycemic control. The findings of the systematic review conducted by Naujokat et al. [14] were similar. Different results were reported in the studies conducted by Eskow and Oates et al. [20], and by Latimer et al. [22], who achieved high rates of dental implant success and survival in patients with diabetes Type II even when their glycemia was poorly controlled. The follow-up in these studies was rather short, however, being only one and two years, respectively.

An important result emerging from a study by Alqahtani et al. [62] lies in that the authors identified a state of chronic hyperglycemia as a stronger mediator of inflammation than cigarette smoking in patients with diabetes mellitus Type II. The systematic literature review and meta-analysis conducted by Monje et al. [86] reported a similar finding, i.e., among non-smokers, those with hyperglycemia had a 3.39-fold risk of developing peri-implantitis compared with individuals with normal blood sugar levels.

Three studies [25,51,63] found that immediately loaded dental implants were just as successful in diabetic patients as in healthy individuals, whereas another study [35] reported an association between immediate loading and a greater probing depth in diabetic patients. Recent systematic reviews and meta-analyses have likewise generated contrasting results [84,87] so it remains impossible to say for sure whether immediately-loaded dental implants are a safe option for diabetic patients.

Two in every three of the studies considered here identified similar rates of implant failure and biological complications in individuals with and without diabetes. The remainder (one in three studies) concluded for diabetes being a risk factor for implant failure, marginal bone loss or biological complications. The literature on the topic is very heterogeneous, partly due to different definitions of peri-implantitis and the use of different clinical indicators of this condition. Most investigators also concentrated on implant failure rather than on peri-implantitis. The results of the present review are similar to those obtained for diabetes in the systematic review conducted by Guobis et al. in 2016 [88].

### 5.2. Obesity

Our literature search revealed only one study (out of a total of six) that found no association between BMI and dental implant failure or late complications [71].

The results reported in three studies [61,68,69] are echoed in a recent meta-analysis [89], which confirmed that bleeding on dental probing was significantly worse in obese patients than in normal-weight individuals. Another study [70] showed that obese patients were at greater risk of localized inflammation involving peri-implant hard, as well as soft, tissues. A study by Vohra et al. [67] identified a significant correlation between serum C-reactive protein (CRP) levels and bleeding on probing, as well as probing depth, in obese patients. This might explain why such patients have worse peri-implant clinical values, though long-term controlled clinical trials would be needed to support these findings (obtained in a retrospective cross-sectional study). Analyzing blood levels of inflammatory molecules could help to identify a possible causal relationship between obesity and peri-implantitis. In cases of obesity, the adipocytes secrete pro-inflammatory cytokines such as TNF-α and IL-6, which stimulate the liver’s production of CRP, altering the hosts’ immune response, and increasing their susceptibility to bacterial infections [11].

Although the above-mentioned studies were consistent in establishing obesity as a risk factor for peri-implantitis, the literature on the topic is still scarce. Hopefully, future research will shed more light on the role of obesity in dental implant rehabilitation.

### 5.3. Cardiovascular Disease

Out of 13 studies selected for review, 11 did not identify cardiovascular disease as a risk factor for dental implant failure or implant-related complications.

Of the two studies that did, one was conducted by Krennmair et al. [21] on a sample of 37 patients: the Authors reported finding a greater bone loss in cases of mandibular full-arch restorations supported by four implants, albeit with a survival rate of 100%. In the other study, Neves et al. [37] found cardiovascular disease associated with a higher implant failure rate, but only hepatitis correlated with a higher risk of peri-implantitis. Their findings were consistent with those of a systematic review conducted by Turri et al. [90].

Given the paucity of research on the topic, it is still impossible to establish a clear link between cardiovascular disease and peri-implantitis. Since inflammation is a condition shared by both diseases, it is important to conduct further research on larger, better-controlled and more homogeneous samples of patients.

### 5.4. Hypertension

Only one study identified higher rates of implant failure in patients with hypertension [48], though the difference was only statistically significant for individuals who were also smokers. AbdulAzeez et al. [53] reported that, in the absence of an adequate monitoring of oral hygiene, systemic diseases had no impact on the severity of bleeding on probing or the probing depth. In fact, the healthy people in their sample revealed more severe inflammation in the oral cavity than patients suffering from diabetes or hypertension. This might be due to an anti-inflammatory effect of antihypertensive medication, which could improve PMN immune cell function [91].

The findings of our review are consistent with a meta-analysis conducted by Schimmel et al. [92], who concluded against hypertension negatively influencing dental implant survival.

### 5.5. Osteoporosis and the Use of Antiresorptive Drugs

Most of the studies examined here (*n* = 16) did not find lower success and survival rates for dental implants in patients with metabolic bone disease than in healthy patients. These results are consistent with the findings of a meta-analysis conducted by Dreyer et al., who found insufficient evidence to claim that osteoporosis is a risk factor for peri-implantitis [85].

As for the use of antiresorptive drugs, most of the studies reviewed did not identify them as risk factors for dental implant rehabilitation [41,46,73,74,75]. The only study that did find such an association [33], also reported 11 cases of implant failure caused by Medication-Related Osteonecrosis of the Jaw (MRONJ) due to sequestering occurring after the removal of an implant.

Some studies identified osteoporosis (*n* = 3) and the use of antiresorptive drugs (*n* = 1) as risk factors for implant success and survival, and for marginal bone loss. One such study by Saminsky et al. [38] concerned just two patients with osteoporosis, who were fitted with a total of nine implants, so this is hardly a representative sample. In another study, Temmerman et al. [81] reported a statistically significant difference in the implant survival rate, but this was due to one patient with osteoporosis requesting the removal of five implants one year after loading—all implants in excellent health in terms of the peri-implant tissues.

It is also important to mention that the study by Alsadi et al. [77] is practically identical to an article previously published by Toy and Uslu [79], as regards the materials and methods, and the results. It was consequently deemed a possible case of plagiarism, and therefore judged unreliable.

In short, the findings of our review concerning osteoporosis and antiresorptive drugs are in line with those of a previous systematic review and meta-analysis by Stavropoulos et al. [93].

## 6. Conclusions

The majority of the studies selected for our literature review covering the last six years consistently conclude against any association between the systemic diseases investigated and late complications in osseo-integrated dental implants. The only exception concerns obesity, which was confirmed as a risk factor in 5/6 studies, though further longitudinal studies with a long follow-up will be needed to confirm even this association. An analysis of the risks of implant complications involving SARS-CoV-2 infection would have made a further contribution from a clinical point of view. However, in agreement with a recent narrative review [94], data linked to peri-implantitis are still missing, and future clinical studies are needed. 

## Figures and Tables

**Table 1:** Articles grouped by findings concerning diabetes as a risk factor for biological complications of dental implants.

Diabetes	Frequencies	% of the Total
Higher risk	17	33.3 %
Risk unchanged	33	64.7 %
Lower risk	1	2.0 %

**Table 2:** Articles grouped by findings concerning obesity as a risk factor for biological complications of dental implants.

Obesity	Frequencies	% of the Total
Higher risk	5	83.3 %
Risk unchanged	1	16.7 %

**Table 3:** Articles grouped by findings concerning cardiovascular disease (CVD) as a risk factor for biological complications of dental implants.

CVD	Frequencies	% of the Total
Higher risk	2	15.4 %
Risk unchanged	11	84.6 %

**Table 4:** Articles grouped by findings concerning hypertension as a risk factor for biological complications of dental implants.

Hypertension	Frequencies	% of the Total
Higher risk	1	14.3 %
Risk unchanged	5	71.4 %
Lower risk	1	14.3 %

**Table 5:** Articles grouped by findings concerning osteoporosis as a risk factor for biological complications of dental implants.

Osteoporosis	Frequencies	% of the Total
Higher risk	3	21.4 %
Risk unchanged	11	78.6 %

**Table 6:** Articles grouped by findings concerning the use of antiresorptive drugs as a risk factor for biological complications of dental implants.

Antiresorptive Drugs	Frequencies	% of the total
Higher risk	1	16.7 %
Risk unchanged	5	83.3 %

## Data Availability

Not applicable.

## Supplementary Materials

The following supporting information can be downloaded at: https://www.mdpi.com/article/10.3390/dj11010010/s1, Table S1. Twenty prospective studies retrieved from the electronic database search; Table S2. Thirty-two retrospective studies retrieved from the electronic database search; Table S3. Thirteen cross-sectional studies retrieved from the electronic database search; and Table S4. Five not specified studies retrieved from the electronic database search.
