# Supplementary material for: Systemic Diseases and Biological Dental Implant Complications: A Narrative Review
Source: Dent J (Basel). 2022 Dec 29;11(1):10. doi: 10.3390/dj11010010 (PMC9857470; doi:10.3390/dj11010010)
Supplement: Supplementary file 1 [file dentistry-11-00010-s001.zip › dentistry-2104549-supplementary.pdf]

SUPPLEMENTARY MATERIALS

Table S1. Twenty prospective studies retrieved from the electronic database search

	TITLE	AUTHOR	YEAR	TYPE OF STUDY	JOURNAL	RISK FACTORS
1	Clinical performance of titanium-zirconium implants with a hydrophilic surface in patients with controlled type 2 diabetes mellitus: 2-year results from a prospective case-control clinical study	José J. Cabrera-Domínguez et al.	2019	Prospective case-control clinical study	Clin Oral Investig	Diabetes
2	Peri-implant soft tissue status and crestal bone levels around adjacent implants placed in patients with and without type-2 diabetes mellitus: 6 years follow-up results	Mohammed N. Alasqah et al.	2018	Prospective clinical study	J Periodontol.	Diabetes
3	A Prospective, Controlled, Multicenter Study to Evaluate the Clinical Outcome of Implant Treatment in Women with Osteoporosis/Osteopenia: 5-Year Results	A. Temmerman et al.	2019	Prospective, Controlled, Multicenter study	J Dent Res	Osteoporosis
4	Assessment of early and late implantation failure of teeth: A single center experience with 297 implanted teeth	Haydar Munil Salih et al.	2021	Prospective, Single center study	AVFT Archivos Venezolanos de Farmacología y Terapéutica	Diabetes
5	Association of Systemic Conditions with Dental Implant Failures in 6,384 Patients During a 31-Year Follow-up Period	Alan B. Carr et al.	2017	Prospective study	Int J Oral Maxillofac Implant	Cardiovascular disease<br>Osteoporosis<br>Diabetes
6	Bone stability around implants in elderly patients with reduced bone mineral density – a prospective study on mandibular overdentures	Luke Chow et al.	2016	Prospective study	Clin Oral Implants Res	Osteoporosis
7	Comparison of clinical and radiographic peri-implant parameters among obese and non-obese patients: A 5-year study	Fahad Alkhudhairy et al.	2018	Longitudinal study	Clin Implant Dent Relat Res	Obesity
8	Implant health and factors affecting peri-implant marginal bone alteration for implants placed in staged maxillary sinus augmentation: A 5-year prospective study	Stefan Krennmair et al.	2019	Prospective study	Clin Implant Dent Relat Res.	Diabetes
	Dental implants treatment outcomes in patient under active therapy with alendronate: 3-year	Marco Tallarico et al.	2016	Prospective observational study	Clin Oral Implants Res	Osteoporosis

9	follow-up results of a multicenter prospective observational study					Alendronate
10	An open, prospective, non-randomized, controlled, multicentre study to evaluate the clinical outcome of implant treatment in women over 60 years of age with osteoporosis/ osteopenia: 1-year results	Andy Temmerman et al.	2016	Open, prospective, non-randomized, controlled, multicentre study	Clin Oral Implants Res	Osteopenia<br>Osteoporosis
11	Dental implant survival and complication rate over two years for individuals with poorly controlled type 2 diabetes mellitus	Caroline C Eskow et al.	2017	Observational study	Clin Implant Dent Relat Res	Diabetes
12	Factors affecting peri-implant bone resorption in four Implant supported mandibular full-arch restorations: a 3-year prospective study	Krennmair S. et al.	2016	Prospective study	J Clin Periodontol	Diabetes<br>Cardiovascular disease
13	Association of Dental Implants Success in Bone Density Classification of Postmenopausal Women with Osteoporosis – A Clinical and Radiographic Prospective Study	Wasim Alsadi et al.	2021	Prospective clinical study	J Indian Acad Oral Med Radiol	Osteoporosis
14	Clinical performance of hydrophilic, titanium-zirconium dental implants in patients with well-controlled and poorly controlled type 2 diabetes: 1-Year results of a dual-center cohort study	Jessica M. Latimer et al.	2021	Dual-center, prospective cohort study	J Periodontol	Poorly controlled diabetes<br>Controlled diabetes
15	Prevalence of periodontitis, dental caries, and peri-implant pathology and their relation with systemic status and smoking habits: Results of an open-cohort study with 22009 patients in a private rehabilitation center	Miguel de Ara´ujo Nobre et al.	2017	Prospective open-cohort study	J Dent	Diabetes<br>Cardiovascular disease
16	Stability and bone loss around submerged and non-submerged implants in diabetic and non-diabetic patients: a 7-year follow-up	Saeed AL ZAHRANI et al.	2018	Prospective observational study	Braz Oral Res	Diabetes
17	One-year performance of posterior narrow diameter implants in hyperglycemic and normo-glycemic patients—a pilot study	Anton Friedmann et al.	2021	Pilot study	Clin Oral Investig	Hyperglycemia

18	Immediate implant-prosthetic dental rehabilitation of patients with diabetes using four immediately loaded dental implants: a pilot study	Raluca-Iulia Juncar et al.	2020	Prospective Clinical Research Report	J Int Med Res	Diabetes
19	Comparison of clinical peri-implant indices and crestal bone levels around narrow and regular diameter implants placed in diabetic and non-diabetic patients: A 3-year follow-up study	Nouf Al-Shibani et al.	2019	Retrospective study	Clin Implant Dent Relat Res	Diabetes
20	Crestal Bone Loss Around Submerged and Non-Submerged Dental Implants in Individuals with Type-2 Diabetes Mellitus: A 7-Year Prospective Clinical Study	Al Zahrani et al.	2019	Prospective study	Med Princ Pract	Poorly controlled diabetes

Table S2. Thirty-two retrospective studies retrieved from the electronic database search

	TITLE	AUTHOR	YEAR	TYPE OF STUDY	JOURNAL	RISK FACTORS
1	Clinical and radiographic indices around narrow diameter implants placed in different glycemic-level patients	Abdulaziz Alsahhaf et al.	2019	Retrospective study	Clin Implant Dent Relat Res	Prediabetes<br>Diabetes
2	Comparison of clinical peri-implant indices and crestal bone levels around narrow and regular diameter implants placed in diabetic and non-diabetic patients: A 3-year follow-up study	Nouf Al-Shibani et al.	2019	Retrospective study	Clin Implant Dent Relat Res	Diabetes
3	Clinical, radiographic, and restorative peri-implant measurements of narrow and standard diameter implants in obese and nonobese patients: A 3-year retrospective follow-up study	Ibraheem F. Alshiddi et al.	2019	Retrospective study	Clin Implant Dent Relat Res	Obesity
4	Comparison of Preprosthetic Implant Complications and Failures Between Obese and Nonobese Patients	Abdel Azeem Hazem et al.	2016	Retrospective study	Int J Oral Maxillofac Implants	Obesity
5	Clinical and Radiographic Peri-Implant Parameters and Whole Salivary Interleukin-1β and Interleukin-6 Levels among Type-2 Diabetic and Nondiabetic Patients with and without Peri-Implantitis	Mansour Al-Askar et al.	2018	Retrospective observational study	Med Princ Pract	Diabetes
	Comparison of peri-implant soft tissue and crestal bone status of dental implants placed in prediabetic, type 2 diabetic,	Abdullah Alshahrani et al.	2020	Retrospective cohort study	Int J Implant Dent	Diabetes

6	and non-diabetic individuals: a retrospective cohort study					
7	Dental Implants with a Calcium Ions-Modified Surface and Platelet Concentrates for the Rehabilitation of Medically Compromised Patients: A Retrospective Study with 5-Year Follow-Up	Marco Mozzati et al.	2021	Retrospective study	Materials (Basel)	Diabetes<br>Osteoporosis
8	Effects of anti-resorptive drugs on implant survival and peri-implantitis in patients with existing osseointegrated dental implants: a retrospective cohort study	J.Y. Kim et al.	2020	Retrospective cohort study	Osteoporos Int	Antiresorptive drugs<br>Diabetes
9	Evaluation of Long-Term Dental Implant Success and Marginal Bone Loss in Postmenopausal Women	VE Toy et al.	2020	Retrospective study	Niger J Clin Pract	Osteoporosis
10	Extensive Autogenous Bone Augmentation and Implantation in Patients Under Bisphosphonate Treatment: A 15-Case Series	Fouad Khoury et al.	2016	Case series	Int J Periodontics Restorative Dent	Bisphosphonates
11	Retrospective Study Of Dental Implants Survival Rate In Postmenopausal Women With Osteoporosis	Wasim Alsaadi et al.	2021	Retrospective study	International Journal of Dentistry and Oral Science (IJDOS)	Osteoporosis
12	Influence of Diabetes on Implant Failure and Peri-Implant Diseases: A Retrospective Study	Alice Alberti et al.	2020	Retrospective study	Dent J (Basel)	Diabetes
13	Dental implants in diabetic patients: retrospective cohort study reporting on implant survival and risk indicators for excessive marginal bone loss at 5 years	M. DE ARAUJO NOBRE et al.	2016	Retrospective cohort study	J Oral Rehabil	Diabetes
14	Relationship Between Osteoporosis and Marginal Bone Loss in Osseointegrated Implants: A 2-Year Retrospective Study	Jose´ R. Corcuera-Flores et al.	2016	Retrospective study	J Periodontol	Osteoporosis
15	Risk indicators for marginal bone resorption around implants in function for at least 4 years: A retrospective longitudinal study	Tomoaki Mameno et al.	2020	Retrospective longitudinal study	J Periodontol	Diabetes<br>Osteoporosis

16	Association between peri-implantitis and cardiovascular diseases: A case-control study	I-Ching Wang et al.	2022	Case control study	J Periodontol	Cardiovascular disease
17	Risk Factors for Implant Failure and Peri-Implant Pathology in Systemic Compromised Patients	Joana Neves et al.	2016	Retrospective clinical study	J Prosthodont	Diabetes<br>Cardiovascular disease
18	Variables Affecting Peri-Implant Radiographic Bone Loss-8-23 Years Follow-Up	Michael Saminsky et al.	2020	Retrospective observational study	Appl. Sci.	Osteoporosis<br>Diabetes<br>Cardiovascular disease
19	Intricate Assessment and Evaluation of Dental Implants in Patients on Bisphosphonate Therapy: A Retrospective Analysis	Suraj Suvarna et al.	2016	Retrospective study	J Contemp Dent Pract	Bisphosphonates
20	Potential risk factors for early and late dental implant failure: a retrospective clinical study on 9080 implants	Henning Staedt et al.	2020	Retrospective clinical study	Int J Implant Dent	Diabetes<br>Cardiovascular disease
21	Retrospective cohort study of 4,591 dental implants: Analysis of risk indicators for bone loss and prevalence of peri-implant mucositis and peri-implantitis	David French et al.	2019	Retrospective cohort study	J Periodontol	Diabetes<br>Bisphosphonates
22	Long term clinical performance of 10 871 dental implants with up to 22 years of follow-up: A cohort study in 4247 patients	David French et al.	2021	Retrospective cohort study	Clin Implant Dent Relat Res	Diabetes
23	The Effect of Controlled Diabetes and Hyperglycemia on Implant Placement with Simultaneous Horizontal Guided Bone Regeneration: A Clinical Retrospective Analysis	Paolo De Angelis et al.	2021	Clinical retrospective study	Biomed Res Int	Diabetes
24	The Effect of Moderately Controlled Type 2 Diabetes on Dental Implant Survival and Peri-implant Bone Loss: A Long-Term Retrospective Study	Zeev Ormianer et al.	2018	Retrospective study	Int J Oral Maxillofac Implants	Diabetes
25	Risk factors associated with short dental implant success: a long-term retrospective evaluation of patients followed up for up to 9 years	HASANOGLU ERBASAR et al.	2019	Multicenter retrospective study	Braz Oral Res	Diabetes type I and II<br>Hypertension
26	Comparative Evaluation of Dental Implant Failure among Healthy and Well-Controlled Diabetic Patients—A 3-Year Retrospective Study	Mohammed Ghazi Sghaireen et al.	2020	Retrospective study	Int J Environ Res Public Health	Diabetes

27	An 11-Year Retrospective Research Study of the Predictive Factors of Peri-Implantitis and Implant Failure: Analytic-Multicentric Study of 1279 Implants in Peru	Frank Mayta-Tovalino et al.	2019	Retrospective multicentric study	Int J Dent	Hypertension<br>Osteoporosis<br>Bisphosphonates<br>Diabetes
28	7-mm-long dental implants: retrospective clinical outcomes in medically compromised patients	Truc Thi Hoang Nguyen et al.	2019	Retrospective study	J Korean Assoc Oral Maxillofac Surg	Diabetes<br>Hypertension
29	A 10 years retrospective study of assessment of prevalence and risk factors of dental implants failures	Rohit Singh et al.	2020	Retrospective study	J Family Med Prim Care	Diabetes<br>Hypertension<br>Cardiovascular disease
30	Assessment of failure rate of dental implants in medically compromised patients	Anuj Singh Parihar et al.	2020	Retrospective study	J Family Med Prim Care	Diabetes<br>Osteoporosis<br>Cardiovascular disease
31	Dental implants in medically complex patients—a retrospective study	Yifat Manor et al.	2016	Retrospective cohort study	Clin Oral Investig	Diabetes<br>Osteoporosis<br>Cardiovascular disease
32	Implant-Supported Immediately Loaded Fixed Full-Arch Dentures: Evaluation of Implant Survival Rates in a Case Cohort of up to 7 Years	Robert Niedermaier et al.	2017	Retrospective 7-years clinical trial	Clin Implant Dent Relat Res	Diabetes<br>Cardiovascular disease<br>Osteoporosis

Table S3. Thirteen cross-sectional studies retrieved from the electronic database search

	TITLE	AUTHOR	YEAR	TYPE OF STUDY	JOURNAL	RISK FACTORS
1	Survival of adjacent-dental-implants in prediabetic and systemically healthy subjects at 5-years follow-up	Mohammed Alrabiah et al.	2018	Cross-sectional clinical study	Clin Implant Dent Relat Res	Prediabetes
2	The Crucial Role of Plaque Control in Peri-Implant Mucositis Initiation as Opposed to the Role of Systemic Health Condition: A Cross-Sectional Study	Ali Raad AbdulAzeez et al.	2021	Cross-sectional study	Clin Cosmet Investig Dent	Diabetes<br>Hypertension

3	Prevalence and risk indicators of peri-implant diseases in a group of Moroccan patients	Jamila Kissa et al.	2020	Cross-sectional study	J Periodontol	Diabetes<br>Cardiovascular disease<br>Hypertension
4	Peri-implant conditions and levels of advanced glycation end products among patients with different glycemic control	Zeyad H. Al-Sowygh et al.	2018	Cross-sectional retrospective study	Clin Implant Dent Relat Res	Diabetes
5	Prevalence of peri-implant diseases among an Italian population of patients with metabolic syndrome: A cross-sectional study	Piero Papi et al.	2019	Cross-sectional study	J Periodontol	Metabolic syndrome
6	Comparison of clinical and radiographic status around immediately loaded versus conventional loaded implants placed in patients with type 2 Diabetes: 12- and 24-month follow-up results	M. D. AL AMRI et al.	2017	Cross-sectional study	J Oral Rehabil	Diabetes
7	Influence of implant location in patients with and without type 2 Diabetes mellitus: 2-year follow-up	T. Abduljabbar et al.	2017	Cross-sectional and retrospective design	Int J Oral Maxillofac Surg	Diabetes
8	Risk indicators for Peri-implantitis. A cross-sectional study with 916 implants	Haline Renata Dalago et al.	2017	Cross-sectional study	Clin Oral Implants Res	Diabetes<br>Cardiovascular disease<br>Hypertension
9	Association of advanced glycation end products with peri-implant inflammation in preDiabetes and type 2 Diabetes mellitus patients	Mohammed Alrabiah et al.	2018	Cross-sectional case-control study	Clin Implant Dent Relat Res	Prediabetes<br>Diabetes
10	Clinical and radiographic peri-implant variables around short dental implants in type 2 diabetic, prediabetic, and non-diabetic patients	Sameer Mokeem et al.	2019	Retrospective cross-sectional study	Clin Implant Dent Relat Res	Bisphosphonates<br>Diabetes
11	Comparison of peri-implant clinical and radiographic inflammatory parameters and whole salivary destructive inflammatory cytokine profile among obese and non-obese men.	Tariq Abduljabbar et al.	2016	Cross-sectional retrospective study	Cytokine	Obesity
12	Peri-implant parameters and C-reactive protein levels amongpatients with different obesity levels	Fahim Vohrar et al.	2018	Cross-sectional retrospective study	Clin Implant Dent Relat Res	Obesity
13	Clinical indices and local levels of inflammatory biomarkers in per-implant health of obese and nonobese individuals	Mohammed N. Alasqah et al.	2018	Clinico-laboratory study	Clin Implant Dent Relat Res	Obesity

Table S4. Five not specified studies retrieved from the electronic database search

	TITLE	AUTHOR	YEAR	TYPE OF STUDY	JOURNAL	RISK FACTORS
1	Clinicoradiographic markers of peri-implantitis in cigarette-smokers and never-smokers with type 2 Diabetes mellitus at 7-years follow-up	Fawaz Alqahtani et al.	2020	Not specified	J Periodontol	Diabetes
2	Comparison of clinical and radiographic parameters around short (6 to 8 mm in length) and long (11 mm in length) dental implants placed in patients with and without type 2 Diabetes mellitus: 3-year follow-up results	Mohammad D. Al Amri et al.	2017	Not specified	Clin Oral Implants Res	Diabetes
3	Comparison of clinical and radiographic status of platform-switched implants placed in patients with and without type 2 Diabetes mellitus: a 24-month follow-up longitudinal study	Mohammad D. Al Amri et al.	2017	Not specified	Clin Oral Implants Res	Diabetes
4	Comparison of clinical and radiographic status around dental implants placed in patients with and without preDiabetes: 1-year follow-up outcomes	Mohammad D. Al Amri et al.	2017	Not specified	Clin Oral Implants Res	Prediabetes
5	Comparison of periodontal and peri-implant inflammatory parameters among patients with preDiabetes, type 2 Diabetes mellitus and non-diabetic controls	Tariq Abduljabbar et al.	2017	Not specified	Acta Odontol Scand	Diabetes<br>Prediabetes
